# Making Antimicrobial Susceptibility Testing More Physiologically Relevant with Bicarbonate?

**DOI:** 10.1128/aac.02412-21

**Published:** 2022-04-18

**Authors:** Mariliis Hinnu, Marta Putrinš, Karin Kogermann, Dirk Bumann, Tanel Tenson

**Affiliations:** a University of Tartu, Institute of Technology, Tartu, Estonia; b University of Tartu, Institute of Pharmacy, Tartu, Estonia; c University of Basel, Biozentrum, Basel, Switzerland

**Keywords:** bicarbonate, azithromycin, pH, *Salmonella*

## Abstract

Azithromycin is a clinically important drug for treating invasive salmonellosis despite poor activity in laboratory assays for MIC. Addition of the main buffer in blood, bicarbonate, has been proposed for more physiologically relevant and more predictive testing conditions. However, we show here that bicarbonate-triggered lowering of azithromycin MIC is entirely due to alkalization of insufficiently buffered media. In addition, bicarbonate is unlikely to be altering efflux pump activity.

## TEXT

Invasive salmonellosis is a major threat to human health affecting >20 million people each year (1, 2). Invasive salmonellosis is caused by Salmonella enterica serovars Typhi, Paratyphi, Enteritidis, or Typhimurium, which all show increasing resistance to previously effective fluoroquinolone and cephalosporin antibiotics. Salmonella strains with such resistances can still be effectively cleared with the macrolide azithromycin (3). The well-documented clinical efficacy of azithromycin is unexpected because recommended doses achieve peak plasma concentrations in the range of only 0.4 mg/L (4), which is 20-fold lower than MIC for the majority of clinical strains of 8 mg/L in standard antimicrobial susceptibility testing (5). However, standard laboratory conditions for susceptibility testing poorly reflect physiological conditions in human tissues (6), thus potentially underestimating Salmonella susceptibility. Indeed, several studies reported that inclusion of the dominant buffer of plasma, bicarbonate HCO_3_^−^, in the assay medium alters the MIC values of many antibiotics, including azithromycin, for diverse bacterial pathogens (Table S1 and references therein, in the supplemental material) (7). Bicarbonate has been proposed to exert these effects by dissipation of the bacterial transmembrane gradient, which results in inactivation of drug efflux pumps (8, 9).

Improving the physiological relevance and accuracy of antimicrobial sensitivity testing is crucial for predicting the therapeutic efficacy of antibiotics against increasingly resistant bacterial pathogens (including emerging Salmonella strains with reduced sensitivity to azithromycin [10]). Bicarbonate might be beneficial in this regard, but its effects could be mediated, at least in part, by trivial pH effects. In aqueous solution, bicarbonate is in equilibrium with gaseous carbon dioxide CO_2_, which can evaporate: CO_2_↑ + H_2_O ⇌ H_2_CO_3_ ⇌ HCO_3_^−^ + H^+^. At low partial pressure of CO_2_, the equilibrium shifts to the left, consuming protons and thus resulting in alkalization (pH increase) of the solution. This could be an important effect since alkaline pH is known to modulate MIC values of various macrolides such as azithromycin (Table S2 and references therein) as well as other antibiotics. To control for this effect, some studies employed 100 mM Tris buffer that should maintain unaltered pH (7, 11), but this has not actually been verified.

**FIG 1:**
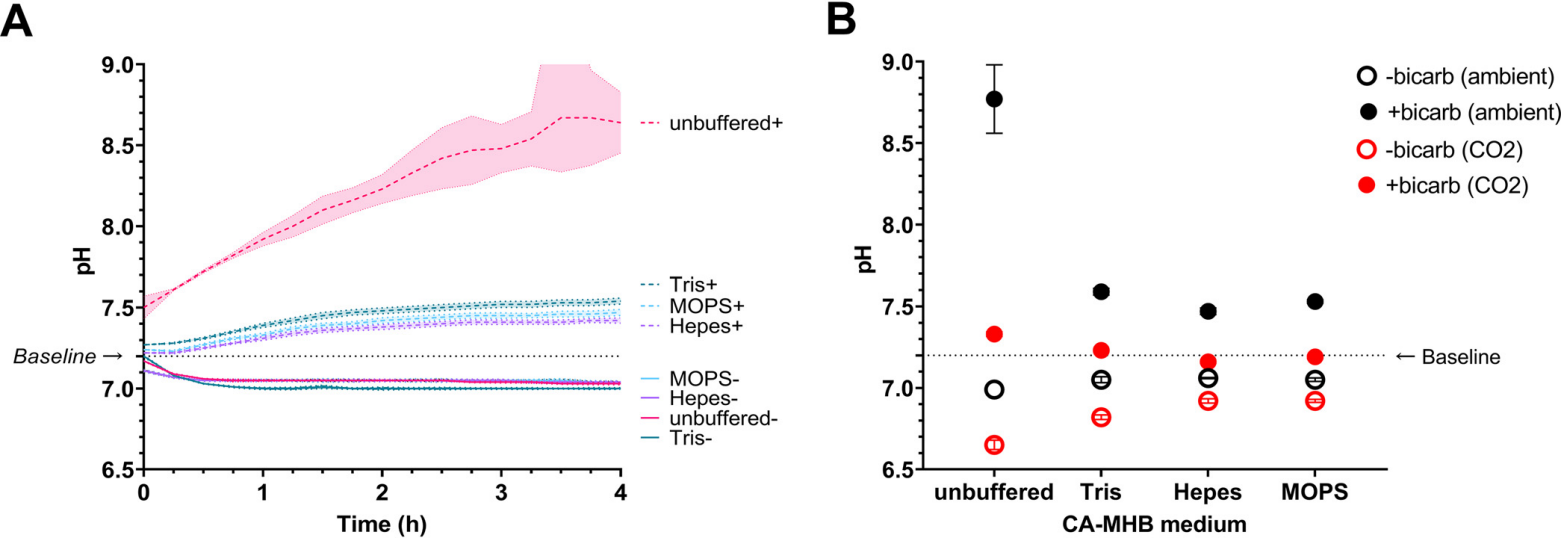
pH changes in cation-adjusted Mueller-Hinton broth with different additions. (A) pH kinetics during incubation in a microtiter plate reader in ambient air (unbuffered or with various buffers at 100 mM; +, addition of 25 mM sodium bicarbonate). pH was determined by ratiometric monitoring of phenol red absorbance at 415 nm and 560 nm. pH values above 8.0 are inaccurate. Arithmetic means and SDs of three independent experiments are shown. (B) pH after 18h of incubation with or without bicarbonate addition (bicarb) in ambient air or a gas atmosphere with 5% CO_2_ (CO2). Arithmetic means and SDs of three independent experiments are shown.

To test the effects of pH and bicarbonate, we monitored pH using the indicator phenol red (Fig. S1). As expected, bicarbonate addition to the standard medium for MIC determination, cation-adjusted Mueller-Hinton broth (CA-MHB), led to an immediate alkalization. We readjusted pH to its initial value 7.2 with HCl followed by sterile filtration and placed the medium in covered multiwell plates that were placed in an incubator at 37°C with ambient air (∼0.3% CO_2_). At this time (*t* = 0 in Fig. 1), the pH had already increased again. Over several hours, the pH rose further to ∼8.5 (Fig. 1A), while pH rose to only 7.33 in the presence of an atmosphere with 5% CO_2_ (Fig. 1B), whereas media acidified, indicating the expected CO_2_ pressure-dependent shift in equilibrium. One hundred mM TRIS and other buffers like MOPS and HEPES partially mitigated, but could not fully prevent, alkalization (pH ∼7.5 after 18 h in ambient air). Buffers together with 5% CO_2_ stabilized best the pH at the desired value of 7.2 when bicarbonate was present (Fig. 1B).

**FIG 2:**
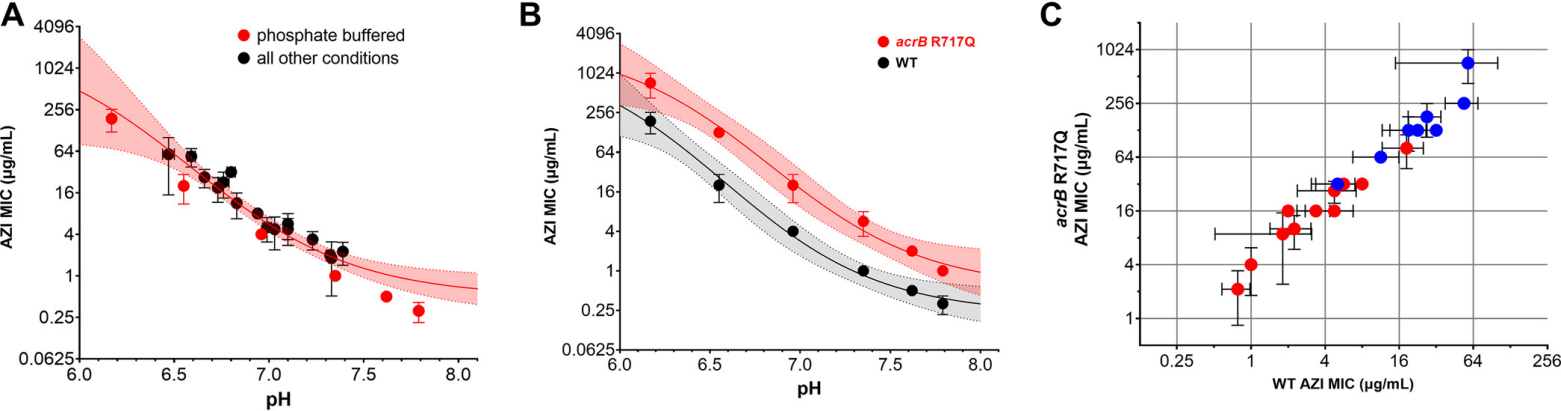
Relationship between MIC values and pH after 18 h of incubation. (A) Relationship between MIC of azithromycin and medium pH for Salmonella enterica serovar Typhimurium. Red circles indicate measurements in phosphate-buffered MHC in ambient air without bicarbonate. Black circles indicate all other experiments (unbuffered, Tris, MOPS, or HEPES buffered; with/without bicarbonate; ambient air/5% CO_2_). Geometric means and SDs (*n* ≥ 3) are shown. The curve and the shaded area represent a nonlinear fit of a Hill function to all experiments except those in phosphate buffer. The shaded area represents the 95% confidence interval. (B) MIC values of Salmonella WT and an *acrB* R717Q mutant with increased efflux activity in phosphate-buffered MHB at various pH (same data for WT as shown in panel A. Geometric means and SDs of three independent experiments are shown. (C) MIC values of Salmonella WT and *acrB* R717Q in all tested conditions (red, with bicarbonate; blue, without bicarbonate). Geometric means and SDs (*n* ≥ 3) are shown.

To quantify the impact of pH shifts, we determined the MIC of wild-type (WT) Salmonella enterica serovar Typhimurium SL1344 (Fig. 2; Table S3). Under standard assay conditions, this strain had MIC values of 4 to 8 μg/mL, indicating susceptibility according to current CLSI breakpoints (S ≤ 16 μg/mL; http://em100.edaptivedocs.net/Login.aspx). Under nonstandard conditions, MIC values varied almost 1,000-fold and inclusion of bicarbonate generally increased Salmonella susceptibility (i.e., lower MIC values). This effect was particularly strong in nonbuffered MHB (>30-fold), which showed the greatest pH increase with bicarbonate, and weaker in MOPS- or HEPES-buffered MHB (∼5-fold), which showed smaller pH shifts (Fig. 1B). Importantly, a plot of all our measured MIC values versus the medium pH after 18 h of incubation followed a common relationship. MHB adjusted to different pH values with phosphate buffer in the absence of bicarbonate and in ambient air yielded superimposable MIC data (Fig. 2A), indicating that pH alone explains the entire “bicarbonate” effect. Visible bacterial growth itself also modified pH with acidification of the medium (Fig. S2). However, the MIC is defined as the lowest concentration at which there is no bacterial growth, and thus MIC values are not affected by growth-associated pH shifts.

For comparison, we introduced an *acrB* R717Q mutation, which increases azithromycin efflux (12). Under standard assay conditions, the mutant had an MIC of 32 μg/mL, indicating resistance according to current CLSI breakpoints (*R* ≥ 32 μg/mL). A ∼5-fold higher MIC compared to wild-type was observed across the entire range of pH 6 to 8 (Fig. 2B and C). This suggested a constant impact of efflux in this pH range. If efflux would instead break down at higher pH, in particular with bicarbonate (as has been proposed), the resistance of the mutant, which is dependent on efflux, should vanish, which is inconsistent with our data. Thus, efflux inactivation does not explain the “bicarbonate effect,” at least for azithromycin and Salmonella. Higher potency of azithromycin at alkaline pH might instead reflect deprotonation of azithromycin’s two amines (13), which might facilitate drug entry through the inner membrane (14, 15).

Development of assay conditions that reflect physiologically relevant conditions for more predictive antimicrobial susceptibility testing remains crucially important. Addition of bicarbonate makes the assay medium more similar to blood in terms of chemical composition, but raises the pH to nonphysiologically high values, unless an atmosphere with 5% CO_2_ is used. If the pH alterations are prevented with the 5% CO_2_ and adequate buffering, bicarbonate has no measurable impact on azithromycin MIC. Thus, bicarbonate addition does not correct a “fundamental flaw” (7) in antimicrobial susceptibility testing.

For azithromycin, the limited available data suggest that standard MIC testing is adequate to predict therapeutic success (5). However, MIC breakpoints differ strongly from achievable plasma levels, suggesting a major difference between inpatient conditions and laboratory assays. Our data show that this discrepancy is not due to bicarbonate. Other explanations, such as intracellular accumulation of azithromycin (16, 17) in the vicinity of Salmonella, seem more plausible, but further research is required to clarify this issue.

## References

[1] Crump JA, Sjölund-Karlsson M, Gordon MA, Parry CM. 2015. Epidemiology, clinical presentation, laboratory diagnosis, antimicrobial resistance, and antimicrobial management of invasive *Salmonella* infections. Clin Microbiol Rev 28:901–937. 10.1128/CMR.00002-15. 26180063 PMC4503790

[2] Marks F, von Kalckreuth V, Aaby P, Adu-Sarkodie Y, El Tayeb MA, Ali M, Aseffa A, Baker S, Biggs HM, Bjerregaard-Andersen M, Breiman RF, Campbell JI, Cosmas L, Crump JA, Espinoza LMC, Deerin JF, Dekker DM, Fields BS, Gasmelseed N, Hertz JT, Van Minh Hoang N, Im J, Jaeger A, Jeon HJ, Kabore LP, Keddy KH, Konings F, Krumkamp R, Ley B, Løfberg SV, May J, Meyer CG, Mintz ED, Montgomery JM, Niang AA, Nichols C, Olack B, Pak GD, Panzner U, Park JK, Park SE, Rabezanahary H, Rakotozandrindrainy R, Raminosoa TM, Razafindrabe TJL, Sampo E, Schütt-Gerowitt H, Sow AG, Sarpong N, Seo HJ, Sooka A, Soura AB, Tall A, Teferi M, Thriemer K, Warren MR, et al. 2017. Incidence of invasive salmonella disease in sub-Saharan Africa: a multicentre population-based surveillance study. Lancet Glob Heal 5:e310–e323. 10.1016/S2214-109X(17)30022-0. PMC5316558 28193398

[3] Dolecek C, La Tran TP, Nguyen NR, Le TP, Ha V, Phung QT, Doan C, Du Nguyen TBB, Duong TL, Luong BH, Nguyen TB, Nguyen TAH, Pham ND, Mai NL, Phan VBB, Vo AH, Nguyen VMH, Tran TTN, Tran TC, Schultsz C, Dunstan SJ, Stepniewska K, Campbell JI, To SD, Basnyat B, Nguyen VVC, Nguyen VS, Nguyen TC, Tran TH, Farrar J. 2008. A multi-center randomised controlled trial of gatifloxacin versus azithromycin for the treatment of uncomplicated typhoid fever in children and adults in Vietnam. PLoS One 3:e2188. 10.1371/journal.pone.0002188. 18493312 PMC2374894

[4] Foulds G, Shepard RM, Johnson RB. 1990. The pharmacokinetics of azithromycin in human serum and tissues. J Antimicrob Chemother 25:73–82. 10.1093/jac/25.suppl_A.73. 2154441

[5] Parry CM, Thieu NTV, Dolecek C, Karkey A, Gupta R, Turner P, Dance D, Maude RR, Ha V, Tran CN, Thi PL, Be BPV, Phi LTT, Ngoc RN, Ghose A, Dongol S, Campbell JI, Thanh DP, Thanh TH, Moore CE, Sona S, Gaind R, Deb M, Anh HV, Van SN, Tinh HT, Day NPJ, Dondorp A, Thwaites G, Faiz MA, Phetsouvanh R, Newton P, Basnyat B, Farrar JJ, Baker S. 2015. Clinically and microbiologically derived azithromycin susceptibility breakpoints for *Salmonella enterica* serovars Typhi and Paratyphi A. Antimicrob Agents Chemother 59:2756–2764. 10.1128/AAC.04729-14. 25733500 PMC4394775

[6] Shi D, Mi G, Wang M, Webster TJ. 2019. *In vitro* and *ex vivo* systems at the forefront of infection modeling and drug discovery. Biomaterials 198:228–249. 10.1016/j.biomaterials.2018.10.030. 30384974 PMC7172914

[7] Ersoy SC, Heithoff DM, Barnes L, Tripp GK, House JK, Marth JD, Smith JW, Mahan MJ. 2017. Correcting a fundamental flaw in the paradigm for antimicrobial susceptibility testing. EBioMedicine 20:173–181. 10.1016/j.ebiom.2017.05.026. 28579300 PMC5478264

[8] Farha MA, French S, Stokes JM, Brown ED. 2018. Bicarbonate alters bacterial susceptibility to antibiotics by targeting the proton motive force. ACS Infect Dis 4:382–390. 10.1021/acsinfecdis.7b00194. 29264917

[9] Farha MA, MacNair CR, Carfrae LA, El Zahed SS, Ellis MJ, Tran HKR, McArthur AG, Brown ED. 2020. Overcoming acquired and native macrolide resistance with bicarbonate. ACS Infect Dis 6:2709–2718. 10.1021/acsinfecdis.0c00340. 32898415

[10] Sajib MSI, Tanmoy AM, Hooda Y, Rahman H, Andrews JR, Garrett DO, Endtz HP, Saha SK, Saha S. 2021. Tracking the emergence of azithromycin resistance in multiple genotypes of typhoidal *Salmonella*. mBio 12:e03481-20. 10.1128/mBio.03481-20. 33593966 PMC8545119

[11] Rose WE, Bienvenida AM, Xiong YQ, Chambers F, Bayer AS, Ersoy SC. 2020. Ability of bicarbonate supplementation to sensitize selected methicillin-resistant *Staphylococcus aureus* strains to β-Lactam antibiotics in an *ex vivo* simulated endocardial vegetation model. Antimicrob Agents Chemother 64:e02072-19. 10.1128/AAC.02072-19. 31844004 PMC7038310

[12] Hooda Y, Sajib MSI, Rahman H, Luby SP, Bondy-Denomy J, Santosham M, Andrews JR, Saha SK, Saha S. 2019. Molecular mechanism of azithromycin resistance among typhoidal *Salmonella* strains in Bangladesh identified through passive pediatric surveillance. PLoS Negl Trop Dis 13:e0007868. 10.1371/journal.pntd.0007868. 31730615 PMC6881056

[13] McFarland JW, Berger CM, Froshauer SA, Hayashi SF, Hecker SJ, Jaynes BH, Jefson MR, Kamicker BJ, Lipinski CA, Lundy KM, Reese CP, Vu CB. 1997. Quantitative structure–activity relationships among macrolide antibacterial agents: *in vitro* and *in vivo* potency against *Pasteurella multocida*. J Med Chem 40:1340–1346. 10.1021/jm960436i. 9135031

[14] Retsema JA, Brennan LA, Girard AE. 1991. Effects of environmental factors on the *in vitro* potency of azithromycin. Eur J Clin Microbiol Infect Dis 10:834–842. 10.1007/BF01975836. 1662627

[15] Butler T, Frenck RW, Johnson RB, Khakhria R. 2001. In vitro effects of azithromycin on *Salmonella typhi*: early inhibition by concentrations less than the MIC and reduction of MIC by alkaline pH and small inocula. J Antimicrob Chemother 47:455–458. 10.1093/jac/47.4.455. 11266420

[16] Wildfeuer A, Laufen H, Zimmermann T. 1996. Uptake of azithromycin by various cells and its intracellular activity under in vivo conditions. Antimicrob Agents Chemother 40:75–79. 10.1128/AAC.40.1.75. 8787883 PMC163060

[17] Hall IH, Schwab UE, Ward ES, Butts JD, Wolford ET, Ives TJ. 2002. Disposition and intracellular activity of azithromycin in human THP-1 acute monocytes. Int J Antimicrob Agents 20:348–360. 10.1016/s0924-8579(02)00187-5. 12431870

