# Supplementary material for: Making Antimicrobial Susceptibility Testing More Physiologically Relevant with Bicarbonate?
Source: Antimicrob Agents Chemother. 2022 Apr 18;66(5):e02412-21. doi: 10.1128/aac.02412-21 (PMC9112938; doi:10.1128/aac.02412-21)
Supplement: Supplemental file 1 — Tables S1 to S3, Fig. S1 and S2, and Text S1. Download aac.02412-21-s0001.pdf, PDF file, 0.3 MB [file aac.02412-21-s0001.pdf]

Supplemental material

Making antimicrobial susceptibility testing more physiologically relevant with bicarbonate?

Mariliis Hinnu, Marta Putrinš, Karin Kogermann, Dirk Bumann, Tanel Tenson

Supplemental tables

Table S1. Previously published papers on the „bicarbonate effect “.

Organism	Antibiotic	Comments on pH	Reference
Various Gram-positive Gram-and negative bacteria	Macrolides:<br>• Azithromycin<br>• Erythromycin	Media (CA-MHB and DMEM) adjusted to pH 7.4 with 100 mM Tris; pH after incubation not determined or discussed	(1)
E. coli<br>*S. aureus*	Macrolides:<br>• Dirithromycin<br>• Erythromycin	CA-MHB medium adjusted to pH 7.4 with HCl;<br>pH after incubation not determined or discussed	(2)
S. aureus MRSA	β-lactams:<br>• Oxacillin<br>• Cefazolin	CA-MHB medium adjusted to pH 7.3 with 100 mM Tris;<br>pH after incubation not determined or discussed	(3–7)
P. aeruginosa<br>*K. pneumoniae*<br>*E .coli, E. cloacae*	Macrolide:<br>• Azithromycin	pH of CA-MHB medium not adjusted	(8)
S. aureus MRSA<br>*S. pneumoniae*<br>*P. aeruginosa*	Macrolide:<br>• Azithromycin	pH of CA-MHB medium not adjusted	(9)
Various Gram-positive Gram-and negative bacteria	Colistin	In some experiments pH of CA-MHB medium adjusted with HCl;<br>pH after incubation not determined	(10)
S. aureus<br>*P. aeruginosa*<br>*E. coli*<br>*S. agalactiae*<br>*E. faecalis*<br>*H. influenza*	no antibiotics	Medium (artificial sputum) without bicarbonate was incubated in ambient air, and with bicarbonate (25 and 100 mM) was incubated in 5% or 20% CO_2 to control pH	(11)<br>(12)

Table S2. Previously published papers on the „pH effect“ on macrolide antibiotics.

Organism	pH effect	Proposed explanation	Reference
H. pylori	macrolides were more active at alkaline pH and less active at acidic pH	-	(13)
H. influenza; E. faealis S. agalactiae; S. aureus S.epidermidis	AZI and 3 other macrolides are more active at alkaline pH and less active at acidic pH. CO_2 incubation will lower the pH of the test medium enough to diminish the potency of macrolides	-	(14)
S.aureus; S.pyogenes E. faecalis; E. coli K. pneumoniae several anaerobes	MIC values for AZI and ERY decrease at higher pH CO_2 incubation will increase MIC due to lower pH	better crossing of the bacterial membrane (unionized AZI) and better binding to the ribosome	(15)
E. coli S. aureus	MIC valuess of erythromycin and roxithromycin for *E. coli* and *S. aureus* were reduced 9-fold and 11-fold, respectively by increasing the pH from 7.2 to 8. Bactericidal activity increased with increasing pH.	-	(16)
Salmonella Typhi from blood cultures of patients	AZI’s MIC decreased stepwise when pH increased with phosphate-citrate and Tris buffers.	Increased transport of AZI into the cells and better access to ribosome at alkaline pH	(17)

Table S3. MIC values of azithromycin for *Salmonella enterica* serovar Typhimurium wild-type (wt) and its *acrB* R717Q mutant (mut)

Buffer	Atmosphere	Bicarbonate addition	Medium pH after 18h	Strain	MIC in µg/mL<br>Range (Geometric mean ± SD)
Unbuffered	Ambient	-	7.11 ± 0.13	wt	4…8<br>(5.0 ± 2.0)
				mut	32<br>(32.0 ± 0.0)
		+	8.77 ± 0.21	wt	0.5…1<br>(0.8 ± 0.2)
				mut	1…4<br>(2.1 ± 1.3)
	5% CO_2	-	6.47 ± 0.03	wt	32…128<br>(58.0 ± 45.3)
				mut	521…1024<br>(724.1 ± 295.6)
		+	7.33 ± 0.0137	wt	1…4<br>(1.8 ± 1.3)
				mut	4-16<br>(8.8 ± 6.4)
	10% CO_2	+	7.32 ± 0.02	wt	2<br>(2.0 ± 0.0)
				mut	16<br>(16.0 ± 0.0)
Tris	Ambient	-	6.8 ± 0.022	wt	32<br>(32.0 ± 0.0)
				mut	128<br>(128.0 ± 0.0)
		+	7.39 ± 0.003	wt	2…4<br>(2.2 ± 0.8)
				mut	8…16<br>(10.1 ± 4.1)
	5% CO_2	-	6.59 ± 0.21	wt	32…64<br>(53.8 ± 16.0)
				mut	256<br>(256.0 ± 0.0)
		+	7.1 ± 0.011	wt	4…8<br>(4.8 ± 2.0)

				mut	16 (16.0 ± 0.0)
Hepes	Ambient	-	6.83 ± 0.003	wt	8...16 (11.3 ± 4.6)
				mut	64 (64.0 ± 0.0)
		+	7.23 ± 0.013	wt	2...4 (3.4 ± 1.0)
				mut	16 (16.0 ± 0.0)
	5% CO_2	-	6.73 ± 0.011	wt	16...32 (19.0 ± 7.4)
				mut	64...256 (128.0 ± 53.4)
		+	7.03 ± 0.009	wt	4...8 (4.8 ± 2.4)
				mut	16...32 (26.9 ± 7.4)
MOPS	ambient	-	6.7 ± 0.029	wt	16...32 (22.6± 9.2)
				mut	128 (128.0 ± 0.0)
		+	7.1 ± 0.009	wt	4...8 (5.7± 2.3)
				mut	32 (32.0 ± 0.0)
	5% CO_2	-	6.66 ± 0.004	wt	16...32 (26.9± 8)
				mut	128...256 (181.0 ± 73.9)
		+	6.94 ± 0.009	wt	8 (8.0 ± 0.0)
				mut	32 (32.0 ± 0.0)
Phosphate pH 6	ambient	-	6.17 ± 0.0062	wt	128...256 (190.2 ± 68.4)
				mut	512...1024 (724.1 ± 295.0)

Phosphate pH 6.5			6.55 ± 0.0025	wt	16...32 (20.2 ± 9.2)
				mut	128 (128.0 ± 0.0)
Phosphate pH 7			6.96 ± 0.0026	wt	4 (4.0 ± 0.0)
				mut	16...32 (20.2 ± 9.24)
Phosphate pH 7.5			7.35 ± 0.0048	wt	1 (1.0 ± 0.0)
				mut	4...8 (5.7 ± 2.3)
Phosphate pH 8			7.62 ± 0.0082	wt	0.5 (0.5 ± 0.0)
				mut	2 (2.0 ± 0.0)
Phosphate pH 8.5			7.79 ± 0.0062	wt	0.25...0.5 (0.3 ± 0.1)
				mut	1 (1.0 ± 0.0)

Supplemental Figures

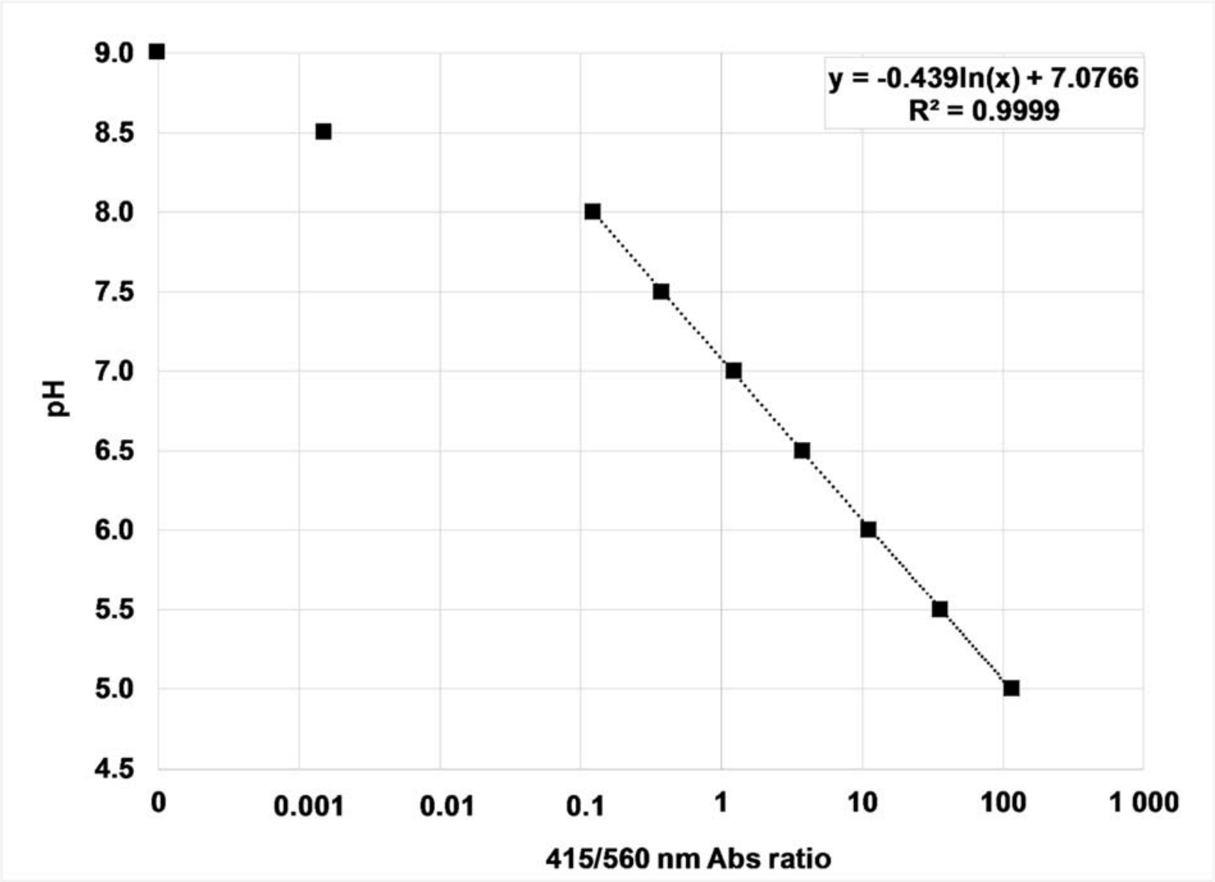

Figure S1. Phenol Red calibration curve for pH in MHB medium without added bicarbonate in ambient air based on ratiometric absorption at 415 and 560 nm. Because of low absorption of phenol red at 415 nm, ratios for pH > 8.0 are not reliable for determining pH. pH was adjusted to a desired value with HCl or NaOH. Arithmetic means of three technical replicates are shown.

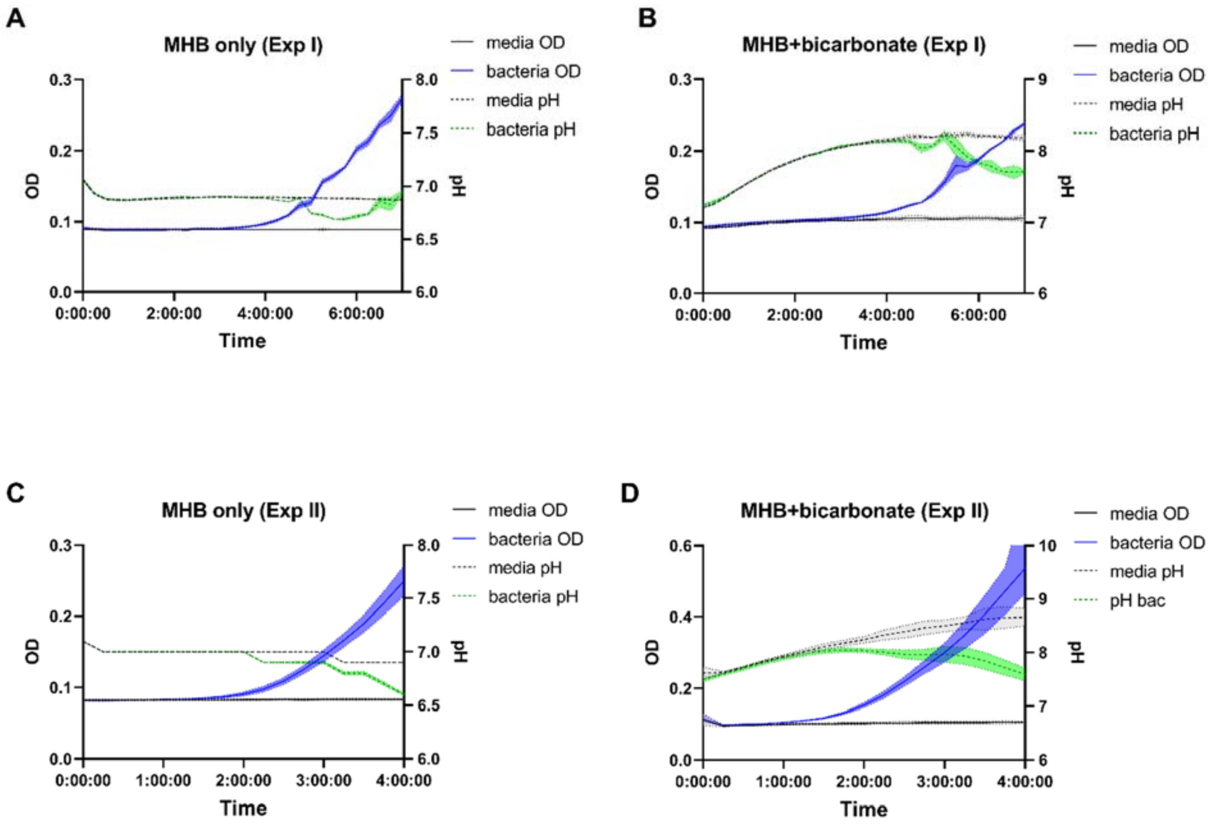

Figure S2. pH kinetics in MHB medium in ambient air with or without *Salmonella* cells and with or without addition of 25 mM sodium bicarbonate. Two independent experiments for each conditions with three or more biological replicates (arithmetic means and SDs) are shown.

Supplemental Text

Materials and methods

Bacterial strains: A prototrophic *hisG*⁺ variant of *Salmonella enterica* serovar Typhimurium SL1344 (18, 19) was used as wild-type. An *acrB* R717Q point mutation associated with reduced azithromycin susceptibility (20) was introduced with a dual-negative selection genome editing method (21). Both strains are resistant to >1 mg/mL streptomycin due to episomal *aph3* and *aph6* genes.

Antibiotic solution: Azithromycin dihydrate (Fluorochem, UK) 15 mg/ml stock solution was prepared in 96% ethanol, and stored at 4°C for up to 7 days. The antimicrobial activity of the azithromycin solution was verified using the quality control strain *Staphylococcus aureus* ATCC 29213 (DSM2569) yielding MIC values of 1-2 ug/mL (6 biological replicates and 3 different media preparations/batches). These values were consistent with the expected range of 0.5-2 ug/mL (http://www.eucast.org).

Buffers & bicarbonate: 1 M buffer stock solutions of Tris(hydroxymethyl)aminomethane hydrochloride (Tris, Sigma), 4-(2-Hydroxyethyl)-1-piperazineethanesulfonic acid (Hepes, Sigma) or 3-Morpholinopropanesulfonic acid (MOPS, Fluka BioChemika) were adjusted to pH 7.4 or 8.1 with HCl or NaOH. To prepare 0.5 M phosphate buffer stock solutions, 0.5 M monobasic sodium phosphate (AppliChem) and 0.5 M dibasic sodium phosphate (AppliChem) were mixed until the desired pH was reached. The buffers and 1 M stock solutions of sodium bicarbonate (Sigma, USA) were sterilized by filtration through 0.22 µm filters and stored at 4°C.

Media: Mueller Hinton II Broth (Cation-Adjusted) (BD®, USA) was used as growth medium. 2X concentrated stock medium was prepared in a Microjet autoclave or by boiling for 1 minute in a microwave oven and filter sterilized in order to assure uniform media composition between experiments. Medium stock was stored at 4°C and used within 2 days. 2X stock medium was filled up to volume with water after addition of bicarbonate (25 mM final concentration) and/or buffer solution (100 mM final concentration), and pH adjustment to 7.2. For the calibration curve, the pH was adjusted with HCl or NaOH to a desired value. Modified media were prepared directly before experiments.

pH measurement with phenol red: Phenol red (0.5% phenol red solution, Sigma) was used at a final concentration of 15 mg/L. Absorbance at 415 nm, 560 nm, and 600 nm (22) was measured with BioTek SynergyMx (BioTek Instruments, Inc., USA) at 37°C in ambient air. Absorbance data were first normalized according to absorbance at 600 nm, then normalized absorbance of media without phenol red was

subtracted, and the 415 nm to 560 nm absorbance ratio was calculated. The results were converted to pH using a calibration curve (Figure S1) and equation

$$pH = -0.9464 * \ln(\text{absorbance ratio}) + 7.007$$

Susceptibility testing: MICs were determined using a standard protocol (23) with 2-fold antibiotic serial dilutions on a 96-well plate in a 150 µl final volume. A standard lid enabling gas exchange with the surrounding atmosphere was used. All incubations were done at 37°C. Plates were incubated statically in ambient air or in a 5% CO_2 incubator. Results were recorded after 18 h by visual inspection. ***Inoculum:*** Strains were streaked from a -80°C glycerol stock onto low-salt lysogeny broth agar containing 90 µg/mL streptomycin and incubated overnight. A single colony was picked for an overnight culture in 3 mL MHB containing 90 µg/ml streptomycin and incubated aerobically. Streptomycin was routinely added in pre-cultures to reduce risks of contamination. On the next day, the culture was 100-fold diluted with fresh MHB without streptomycin and grown until OD_{600} 0.5 to 0.8. The cultures were then diluted to OD 0.1 in unmodified MHB medium, followed by further dilution in various media to an initial density of 3 – 7 x 10^5 CFU/mL, which was verified by plating.

Supplementary References

1. **Ersoy SC**, **Heithoff DM**, **Barnes L**, **Tripp GK**, **House JK**, **Marth JD**, **Smith JW**, **Mahan MJ**, **Mahan MJ**. 2017. Correcting a Fundamental Flaw in the Paradigm for Antimicrobial Susceptibility Testing. EBioMedicine **20**:173–181.

2. **Farha MA**, **French S**, **Stokes JM**, **Brown ED**. 2018. Bicarbonate Alters Bacterial Susceptibility to Antibiotics by Targeting the Proton Motive Force. ACS Infect Dis **4**:382–390.

3. **Ersoy SC**, **Abdelhady W**, **Li L**, **Chambers HF**, **Xiong YQ**, **Bayer AS**. 2019. Bicarbonate Resensitization of Methicillin-Resistant Staphylococcus aureus to β-Lactam Antibiotics. Antimicrob Agents Chemother **63**:e00496-19.

4. **Rose WE**, **Bienvenida AM**, **Xiong YQ**, **Chambers HF**, **Bayer AS**, **Ersoy SC**. 2020. Ability of Bicarbonate Supplementation To Sensitize Selected Methicillin-Resistant Staphylococcus aureus Strains to β-Lactam Antibiotics in an Ex Vivo Simulated Endocardial Vegetation Model. Antimicrob Agents Chemother **64**:e02072-19.

5. **Ersoy SC**, **Otmishi M**, **Milan VT**, **Li L**, **Pak Y**, **Mediavilla J**, **Chen L**, **Kreiswirth B**, **Chambers HF**, **Proctor RA**, **Xiong YQ**, **Fowler VG**, **Bayer AS**. 2020. Scope and Predictive Genetic/Phenotypic Signatures of Bicarbonate ($NaHCO_3$) Responsiveness and β-Lactam Sensitization in Methicillin-Resistant Staphylococcus aureus. Antimicrob Agents Chemother **64**:e02445-19.

6. **Ersoy SC**, **Rose WE**, **Patel R**, **Proctor RA**, **Chambers HF**, **Harrison EM**, **Pak Y**, **Bayer AS**. 2021. A Combined Phenotypic-Genotypic Predictive Algorithm for In Vitro Detection of Bicarbonate: β-Lactam Sensitization among Methicillin-Resistant Staphylococcus aureus (MRSA). Antibiot 2021, Vol 10, Page 1089 **10**:1089.

7. **Ersoy SC**, **Chambers HF**, **Proctor RA**, **Rosato AE**, **Mishra NN**, **Xiong YQ**, **Bayer AS**. 2021. Impact of bicarbonate on PBP2A production, maturation, and functionality in methicillin-resistant staphylococcus aureus. Antimicrob Agents Chemother **65**.

8. **Meerwein M**, **Tarnutzer A**, **Böni M**, **Van Bambeke F**, **Hombach M**, **Zinkernagel AS**. 2020. Increased Azithromycin Susceptibility of Multidrug-Resistant Gram-Negative Bacteria on RPMI-1640 Agar Assessed by Disk Diffusion Testing. Antibiotics.

9. **Farha MA**, **MacNair CR**, **Carfrae LA**, **El Zahed SS**, **Ellis MJ**, **Tran H-KR**, **McArthur AG**, **Brown ED**. 2020. Overcoming Acquired and Native Macrolide Resistance with Bicarbonate. ACS Infect Dis **6**:2709–2718.

10. **Panta PR**, **Doerrler WT**. 2021. A link between pH homeostasis and colistin resistance in bacteria. Sci Rep **11**:1–16.

11. **Jaikumpun P**, **Ruksakiet K**, **Stercz B**, **Pállinger É**, **Steward M**, **Lohinai Z**, **Dobay O**, **Zsembery Á**. 2020. Antibacterial Effects of Bicarbonate in Media Modified to Mimic Cystic Fibrosis Sputum. Int J Mol Sci 2020, Vol 21, Page 8614 **21**:8614.

12. **Dobay O**, **Laub K**, **Stercz B**, **Kéri A**, **Balázs B**, **Tóthpál A**, **Kardos S**, **Jaikumpun P**, **Ruksakiet K**, **Quinton PM**, **Zsembery Á**. 2018. Bicarbonate inhibits bacterial growth and biofilm formation of prevalent cystic fibrosis pathogens. Front Microbiol **9**:2245.

13. **Hardy DJ**, **Hanson CW**, **Hensey DM**, **Beyer JM**, **Fernandes PB**. 1988. Susceptibility of Campylobacter pylori to macrolides and fluoroquinolones. J Antimicrob Chemother **22**:631–636.

14. **Barry AL**, **Jones RN**, **Thornsberry C**. 1988. In vitro activities of azithromycin (CP 62,993), clarithromycin (A-56268; TE-031), erythromycin, roxithromycin, and clindamycin. Antimicrob Agents Chemother **32**:752 LP – 754.

15. **Retsema JA**, **Brennan LA**, **Girard AE**. 1991. Effects of environmental factors on the in vitro potency of azithromycin. Eur J Clin Microbiol Infect Dis **10**:834–842.

16. **Pruul H**, **McDonald PJ**. 1992. Potentiation of antibacterial activity of azithromycin and other macrolides by normal human serum. Antimicrob Agents Chemother **36**:10–16.

17. **Butler T**, **Frenck RW**, **Johnson RB**, **Khakhria R**. 2001. In vitro effects of azithromycin on Salmonella typhi: early inhibition by concentrations less than the MIC and reduction of MIC by alkaline pH and small inocula. J Antimicrob Chemother **47**:455–458.

18. **Hoiseth SK**, **Stocker BA**. 1981. Aromatic-dependent Salmonella typhimurium are non-virulent and effective as live vaccines. Nature **291**:238–239.

19. **Claudi B**, **Spröte P**, **Chirkova A**, **Personnic N**, **Zankl J**, **Schürmann N**, **Schmidt A**, **Bumann D**. 2014. Phenotypic variation of Salmonella in host tissues delays eradication by antimicrobial chemotherapy. Cell **158**:722–33.

20. **Hooda Y**, **Sajib MSI**, **Rahman H**, **Luby SP**, **Bondy-Denomy J**, **Santosham M**, **Andrews JR**, **Saha SK**, **Saha S**. 2019. Molecular mechanism of azithromycin resistance among typhoidal Salmonella strains in Bangladesh identified through passive pediatric surveillance. PLoS Negl Trop Dis **13**:e0007868.

21. **Cianfanelli FR**, **Cunrath O**, **Bumann D**. 2020. Efficient dual-negative selection for bacterial genome editing. BMC Microbiol **20**:129.

22. **Schornack PA**, **Gillies RJ**. 2003. Contributions of Cell Metabolism and H+ Diffusion to the Acidic pH of Tumors. Neoplasia **5**:135–145.

23. **EUCAST**. 2003. Determination of minimum inhibitory concentrations (MICs) of antibacterial agents by broth dilution. Clin Microbiol Infect **9**:ix–xv.
